# Deep learning for anatomical interpretation of video bronchoscopy images

**DOI:** 10.1038/s41598-021-03219-6

**Published:** 2021-12-09

**Authors:** Ji Young Yoo, Se Yoon Kang, Jong Sun Park, Young-Jae Cho, Sung Yong Park, Ho Il Yoon, Sang Jun Park, Han-Gil Jeong, Tackeun Kim

**Affiliations:** 1 Department of Anesthesiology and Pain Medicine, Ajou University School of Medicine, Suwon, South Korea; 2 Division of Pulmonary and Critical Care Medicine, Department of Internal Medicine, Seoul National University Bundang Hospital, Seongnam, South Korea; 3 Seoul National University College of Medicine, Seoul, South Korea; 4 Department of Ophthalmology, Seoul National University Bundang Hospital, Seongnam, South Korea; 5 Department of Neurosurgery, Seoul National University Bundang Hospital, 82 Gumi-ro 173 beon-gil, Bundang-gu,, Seongnam, 13620 South Korea; 6 Department of Neurology, Seoul National University Bundang Hospital, Seongnam, South Korea; 7 TALOS Corp., Yongin, South Korea

**Keywords:** Anatomy, Medical research, Computational models

## Abstract

Anesthesiologists commonly use video bronchoscopy to facilitate intubation or confirm the location of the endotracheal tube; however, depth and orientation in the bronchial tree can often be confused because anesthesiologists cannot trace the airway from the oropharynx when it is performed using an endotracheal tube. Moreover, the decubitus position is often used in certain surgeries. Although it occurs rarely, the misinterpretation of tube location can cause accidental extubation or endobronchial intubation, which can lead to hyperinflation. Thus, video bronchoscopy with a decision supporting system using artificial intelligence would be useful in the anesthesiologic process. In this study, we aimed to develop an artificial intelligence model robust to rotation and covering using video bronchoscopy images. We collected video bronchoscopic images from an institutional database. Collected images were automatically labeled by an optical character recognition engine as the carina and left/right main bronchus. Except 180 images for the evaluation dataset, 80% were randomly allocated to the training dataset. The remaining images were assigned to the validation and test datasets in a 7:3 ratio. Random image rotation and circular cropping were applied. Ten kinds of pretrained models with < 25 million parameters were trained on the training and validation datasets. The model showing the best prediction accuracy for the test dataset was selected as the final model. Six human experts reviewed the evaluation dataset for the inference of anatomical locations to compare its performance with that of the final model. In the experiments, 8688 images were prepared and assigned to the evaluation (180), training (6806), validation (1191), and test (511) datasets. The EfficientNetB1 model showed the highest accuracy (0.86) and was selected as the final model. For the evaluation dataset, the final model showed better performance (accuracy, 0.84) than almost all human experts (0.38, 0.44, 0.51, 0.68, and 0.63), and only the most-experienced pulmonologist showed performance comparable (0.82) with that of the final model. The performance of human experts was generally proportional to their experiences. The performance difference between anesthesiologists and pulmonologists was marked in discrimination of the right main bronchus. Using bronchoscopic images, our model could distinguish anatomical locations among the carina and both main bronchi under random rotation and covering. The performance was comparable with that of the most-experienced human expert. This model can be a basis for designing a clinical decision support system with video bronchoscopy.

## Introduction

In this era of artificial intelligence, clinical decision support systems have been developed using artificial intelligence and used to mitigate physicians’ effort and improve patient outcomes^1–4^. To build a reliable and robust system, well-trained algorithms with enormous amount of thoroughly prepared dataset are required to obtain reliable performance.

Video bronchoscopy is an important tool for airway inspections^5^. Generally, anatomical discrimination of the bronchial tree during diagnostic video bronchoscopy examinations is achieved by tracing the airway from the oral space to the deeper bronchi. In addition, navigation bronchoscopy has been developed to support examination process^6–10^. In anesthesia, video bronchoscopy is commonly used to intubate difficult airways and to confirm the proper positioning of lung-isolation devices, such as the double-lumen tube or endobronchial blocker^11–16^. Thus, an accurate understanding and knowledge of bronchial tree anatomy are essential for an anesthesiologist when using video bronchoscopy^16,17^. Unlike general diagnostic video bronchoscopic procedures, anesthesiologists often cannot determine anatomical locations by tracing from the oral cavity to the deeper bronchi when it is performed using an endotracheal tube. Moreover, the position of patients according to operations (e.g., lateral decubitus position) can cause confusion with respect to the orientation of bronchoscopic view, and the bronchial part of the double-lumen tube or bronchial blocker often blocks the view. Thus, it is more difficult to determine the depth and location in the bronchial tree in anesthesiologic procedures than in routine diagnostic video bronchoscopy. Although rare, misinterpretation of tube location can cause accidental extubation or endobronchial intubation, which can lead to complications, such as atelectasis in the unventilated side and barotrauma of the intubated side^18,19^.

In this study, we developed artificial intelligence model robust to rotation and covering for anatomical interpretation of video bronchoscopy images which can be a useful option in anesthesiologic process.

## Materials and methods

### Data preparation and preprocessing

The retrospective data collection and analysis plan was approved by the Institutional Review Board of the Seoul National University Bundang Hospital and the need for obtaining informed consent was waived (B-2001/588-102). All research processes were performed in accordance with the Declaration of Helsinki. We subsequently searched the clinical data warehouse of our institution for patients who had undergone video bronchoscopy.

Because some information regarding anatomical location was missing since 2008, we limited the scope of search from 2004 to 2007. A total of 3216 patients underwent video bronchoscopy from January 2004 to December 2007. Through the picture archiving and communication system, we could download 47,447 images containing text annotations regardless of age, sex, and diagnosis.

Collected images were automatically labeled using an open-source optical character recognition engine (Tesseract, version 4.1.1, https://tesseract-ocr.github.io). To enhance optical character recognition performance, we converted the color images to gray and then applied binary thresholding using the OpenCV library (version 4.4.0, https://opencv.org). If extraction of meaningful strings from images of the original size was not possible, we attempted to extract strings from images sequentially magnified by 2–10 times. All recognized text strings were converted to lowercase. If any text containing “car” was found, that image was assigned to the carina class. In a similar manner, images with text containing “left main”, “lt. main”, or “lm” were assigned to the left main bronchus class and images containing “right main”, “rt. main”, or “rm” were assigned to the right main bronchus class. Images which represented anatomical position other than the carina and main bronchi or could not be identified by automated labeling were excluded (37,654). The remaining 9793 images were successfully placed in the carina class (3228), left main bronchus class (3471) and right main bronchus class (3094).

Next, a single researcher (TK) evaluated the entire image set manually and excluded images with a foreign body, tumors, massive sputum, or hemorrhage blocking normal anatomical structures. Images showing traces of surgery or very poor quality were also excluded. Consequently, 1105 inappropriate images were discarded by manual evaluation. Finally, 8688 images were prepared for experiments (3100 for the carina class, 2901 for the left main bronchus class, and 2687 for the right main bronchus class). For experimental images, only a squared area containing the bronchoscopic view was cropped, and the rest of the canvas, including patient-related information, was removed. Finally, all images were resized to 224 by 224 pixels.

**Figure 1:**
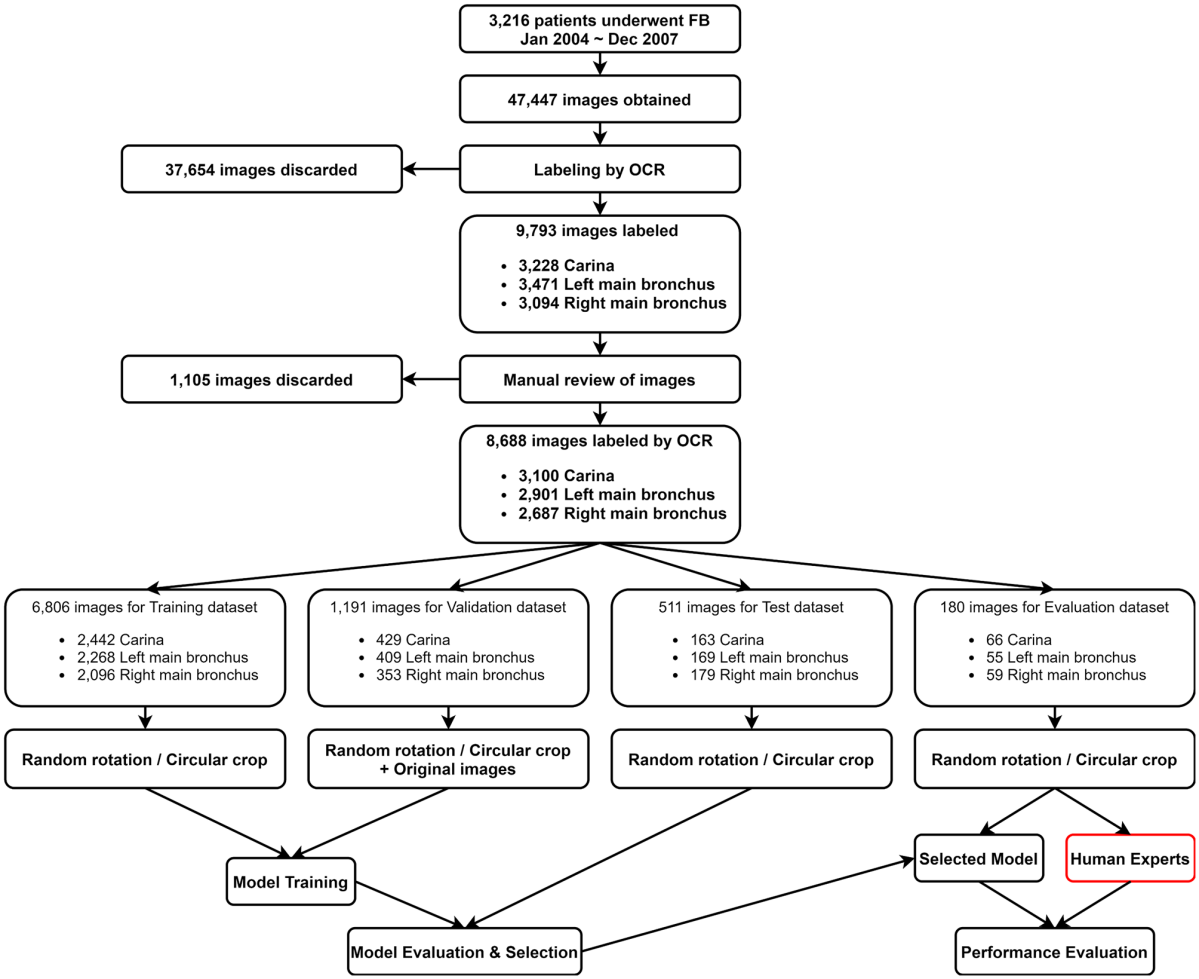
Image preprocessing. Images were labeled automatically by an optical character recognition engine. Recognized text strings were classified as the carina, left main bronchus, and right main bronchus classes. Only a square area containing the bronchoscopic view was cropped, and the rest, including patient-related information, was removed. All images were resized to 224 by 224 pixels and randomly rotated and cropped to a circle of random radius.

Prepared images were categorized into four datasets using a random permutation: training dataset (used for model training), validation dataset (used for model training for calculating validation accuracy and loss), test dataset (used for evaluating each experiment to select the best model), and evaluation dataset (used for comparing model performance to that of human experts). First, 180 images were selected and isolated for the evaluation dataset. Then, 80% (6806) of the remaining 8,508 images were randomly allocated to the training dataset and the remaining 1702 were randomly divided in a 7:3 ratio and assigned to the validation dataset (1191) and test dataset (511), respectively (Fig. 1).

**Figure 2:**
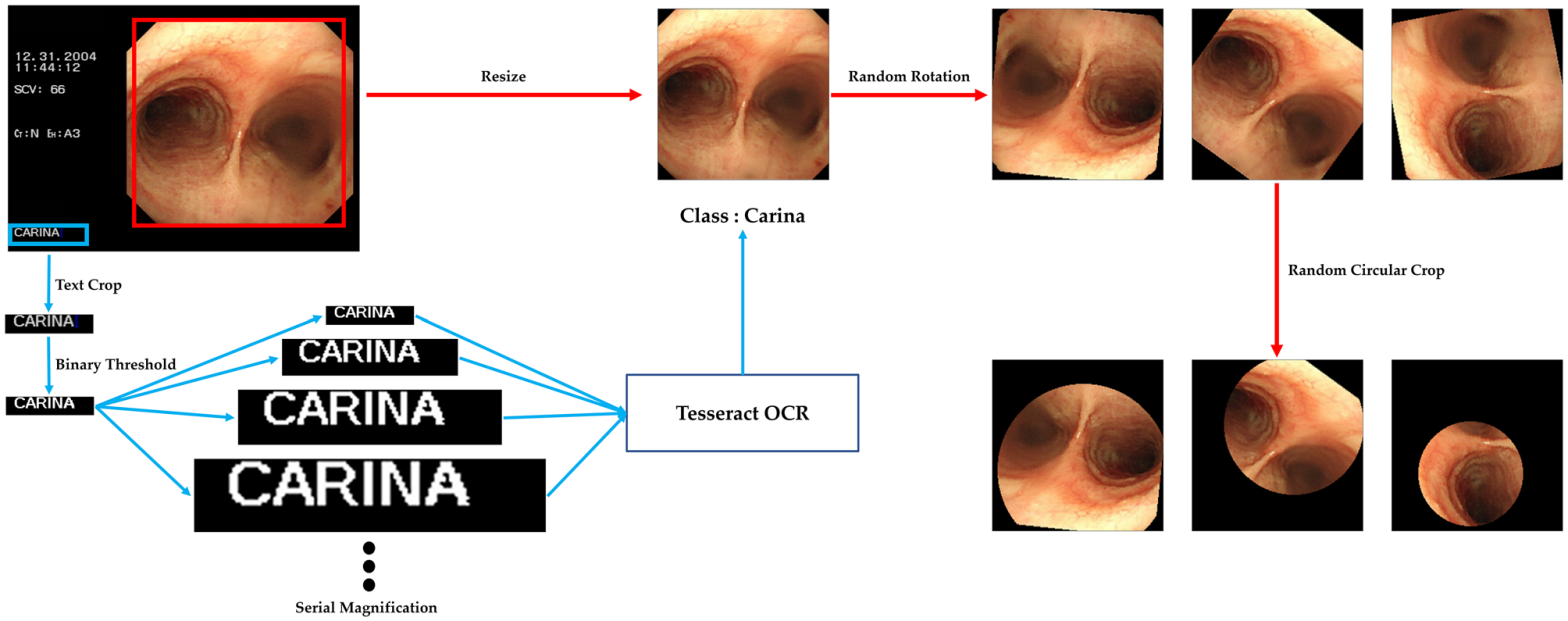
Data preparation and partitioning process.

The training, test, and evaluation datasets were randomly rotated (0–2π) and cropped to a circle of random radius from 60 to 112 pixels, where the x–y coordinates of the center point between 72 and 152 were randomly assigned, respectively, to make the model robust to rotation and covering by the endotracheal tube. Regarding the validation dataset, the same preprocessing was applied, but the original images were also appended to optimize the model training process to enhance the classification performance (Fig. 2).

### Model training and evaluation

We used TensorFlow (version 2.3.1, https://www.tensorflow.org) as a back-end library on the Python (version 3.6.9, https://www.python.org) programming language. To search for adequate models for our classification problem, we adopted pretrained convolutional neural network models provided as application programming interfaces by the TensorFlow library. Models with < 25 million parameters were selected considering training and inference efficiency for the possibility of being embedded in endoscopic equipment in the future. Thus, 10 models (DenseNet121, DenseNet201, EfficientNetB0, EfficientNetB1, EfficientNetB2, EfficientNetB3, EfficientNetB4, MobileNetV2, NASNetMobile, and ResNet50V2) with pretrained weights with Imagenet dataset were adopted for this investigation^20–25^.

Using each pretrained model, several modifications were made to fit our classification task. First, the shape of the input array was set to (224, 224, 3) for models with different input shape. Next, the output layer containing 1000 fully connected nodes was replaced with three fully connected nodes activated by a normalized exponential function (softmax) to predict ternary classes of our datasets. Loss function was defined with categorical cross-entropy. An Adam optimizer was used^26^. Batch size was equally set to 128 for all models considering the maximum parameter numbers and size of the graphics-processing unit memory (32 GB × 2). As to the initial learning rate, we performed grid search to identify the best parameter among 10^–2^, 10^–4^, 10^–6^, and 10^–8^. While monitoring the loss function for the validation dataset, we proceeded with training to minimize the loss function. If the minimum loss was not updated during five epochs, the learning rate was reduced by 0.9. In the case of failure to update the lowest loss value for 100 epochs, training was terminated, and the saved model with the lowest loss was used as the best model of each experiment. The same training processes were applied to all 10 models.

Model evaluation was performed by using a test dataset after all model training processes were finished. Using each best model, the prediction of classes and related probabilities was inferred to calculate the numbers of true positives, true negatives, false positives, and false negatives. The best prediction accuracy of a model defined as $\frac{\text{TP}\,+\,\text{TN}}{\text{TP}\,+\,\text{TN}\,+\,\text{FP}\,+\,\text{FN}}$ was selected as the final artificial intelligence model. Using the artificial intelligence model, the area under the receiver operating characteristic curve (AUC) and area under the precision $\left(\frac{\text{TP}}{\text{TP}\,+\,\text{FP}}\right)$ − recall $\left(\frac{\text{TP}}{\text{TP}\,+\,\text{FN}}\right)$ curve were plotted for each class.

### Performance comparison with human experts

To evaluate and compare model performance against human experts, 200 images were prepared from 180 isolated evaluation dataset images; 20 were randomly selected and added to measure test–retest reliability. Each of the 200 images underwent random rotation and circular crop in the manner described above, and true labels were blinded. Three anesthesiologists (A1, A2, and A3) with 1, 15, and 24 years, respectively, of specialist experience and three pulmonologists (P1, P2, and P3) with 12, 14, and 20 years, respectively, of specialist experience at a referral university hospital reviewed 200 images to infer anatomical locations. Inference results were also obtained by substituting the same image set for the artificial intelligence model.

For each evaluator, including the artificial intelligence model, two methods were used to measure the performance: the first to measure performance for the originally planned ternary classification and the second to measure performance for binary classification distinguishing the carina from both bronchi. In both methods, classification accuracy, precision, and recall were individually calculated.

### Explanation of artificial intelligence model

The mode of action of a convolutional neural network as a classifier is difficult to intuitively understand. Thus, several methods have been introduced for visualization of the decision basis. Among them, we adopted gradient-weighted class activation mapping (Grad-CAM), which could be calculated by average pooling of weights formed by each convolution layer for visualization of the anatomical structures that influence the prediction^27^.

### Statistical analysis

The statsmodels library (version 0.12.1, https://statsmodels.org) for Python was used. The chi-square test was applied to compare the proportions of classes among datasets. To compare classification performance, the McNemar test was performed by using paired answers between evaluators. p-value of < 0.05 was considered statistically significant.

## Results

There was no significant difference in the class distribution among the four datasets (training, validation, test, and evaluation datasets) (X2 = 6.6487, p = 0.3546).

**Figure 3:**
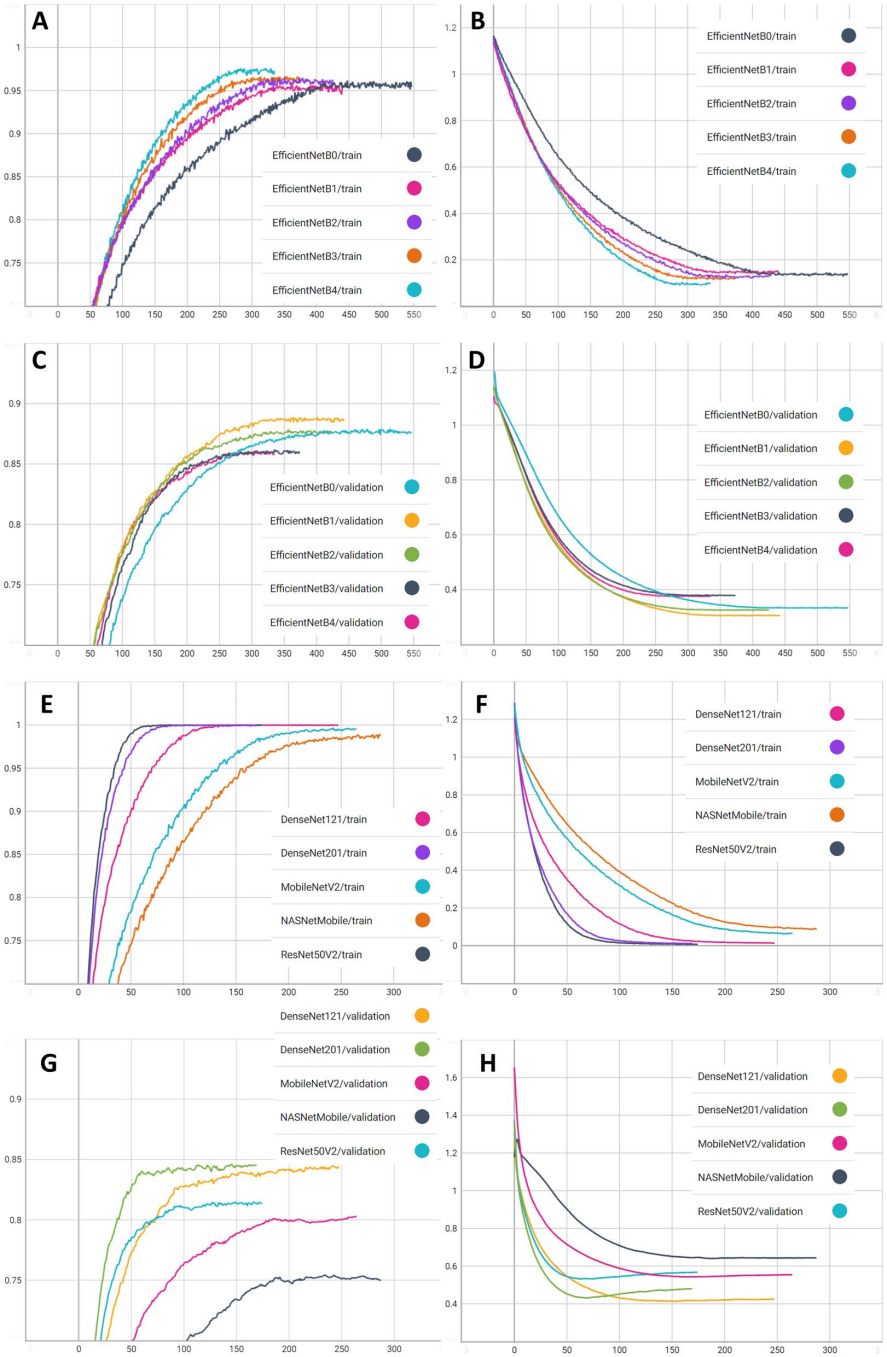
Performance metrics changes during the training process of each base model using a learning rate of 10^−6^. The horizontal axis represents the number of epochs. (**A**) training accuracies for the EfficientNet family; (**B**) training losses for the EfficientNet family; (**C**) validation accuracies for the EfficientNet family; (**D**) validation losses for the EfficientNet family; (**E**) training accuracies for models other than the EfficientNet family; (**F**) training losses for models other than the EfficientNet family; (**G**) validation accuracies for models other than the EfficientNet family; (**H**) validation losses for models other than the EfficientNet family.

The results of the training process using the base model adopted for custom model construction fit to our task are summarized in Table 1. Generally, the training process using a learning rate of 10^–4^ showed faster convergence, while using a learning rate of 10^–6^ showed higher accuracy for the validation dataset. In most cases, parameters could not be converged with a learning rate of 10^–8^. The DenseNet201 based model with a learning rate of 10^–4^ showed the lowest loss value (0.2039) for the validation dataset. However, the highest accuracy for the validation dataset was achieved by EfficientNetB1 based model trained with a learning rate of 10^–6^ (0.8871). Figure 3 shows the change in performance metrics during the training process with a learning rate of 10^–6^ according to each base model. Using models showed the best accuracy for validation dataset by each base model, accuracy for test dataset was measured to select the final model. The model based on EfficientNetB1 trained using a learning rate of 10^–6^ showed the highest accuracy (0.8630) for the test dataset; the precision and recall were 0.8661 and 0.8652, respectively, for the test dataset. Thus, the EfficientNetB1 based model trained with a learning rate of 10^–6^ was selected as the artificial intelligence model.

**Table 1:** Training results according to the base models used for custom model design and initial learning rate.

Base model	Number of parameters	Model size (MB)	Learning rate	Training epochs	Loss for validation dataset	Accuracy for validation dataset	Accuracy for test dataset
DenseNet121	7,040,579	82	10^–2^	72	0.5139	0.7322	
			10^–4^	46	0.2269	0.7649	
			10^–6^	147	0.4132	0.8375	0.8141
			10^–8^	Failed to converge			
DenseNet201	18,327,747	212	10^–2^	54	0.5457	0.7200	
			10^–4^	32	0.2039*	0.7720	
			10^–6^	69	0.4307	0.8380	0.8239
			10^–8^	2205	0.6662	0.7364	
EfficientNetB0	4,053,414	48	10^–2^	52	0.4440	0.8296	
			10^–4^	6	0.2841	0.8098	
			10^–6^	497	0.3324	0.8787	0.8434
			10^–8^	Failed to converge			
EfficientNetB1	6,579,082	77	10^–2^	81	0.3607	0.8438	
			10^–4^	5	0.2641	0.8237	
			10^–6^	392	0.3047	0.8871^†^	0.8630^‡^
			10^–8^	Failed to converge			
EfficientNetB2	7,772,796	90	10^–2^	51	0.3224	0.8375	
			10^–4^	4	0.2984	0.8014	
			10^–6^	375	0.3248	0.8774	0.8589
			10^–8^	Failed to converge			
EfficientNetB3	10,788,146	125	10^–2^	65	0.4671	0.8119	
			10^–4^	4	0.3046	0.8077	
			10^–6^	323	0.3781	0.8610	0.8258
			10^–8^	Failed to converge			
EfficientNetB4	17,679,202	204	10^–2^	55	0.3126	0.8308	
			10^–4^	31	0.3089	0.8338	
			10^–6^	285	0.3751	0.8589	0.8434
			10^–8^	Failed to converge			
MobileNetV2	2,261,827	27	10^–2^	235	0.4963	0.7397	
			10^–4^	103	0.5407	0.7208	
			10^–6^	164	0.5426	0.7926	0.7789
			10^–8^	4026	0.8471	0.6121	
NASNetMobile	4,272,887	53	10^–2^	858	0.7217	0.8237	
			10^–4^	166	0.4902	0.8799	0.8219
			10^–6^	187	0.6392	0.7519	
			10^–8^	Failed to converge			
ResNet50V2	23,570,947	271	10^–2^	25	0.5966	0.6377	
			10^–4^	23	0.2962	0.7531	
			10^–6^	74	0.5310	0.8023	0.7808
			10^–8^	1902	0.7737	0.6688	

*MB* megabytes.

*The lowest loss value.

^†^The highest accuracy for validation dataset.

^‡^The highest accuracy for test dataset.

**Figure 4:**
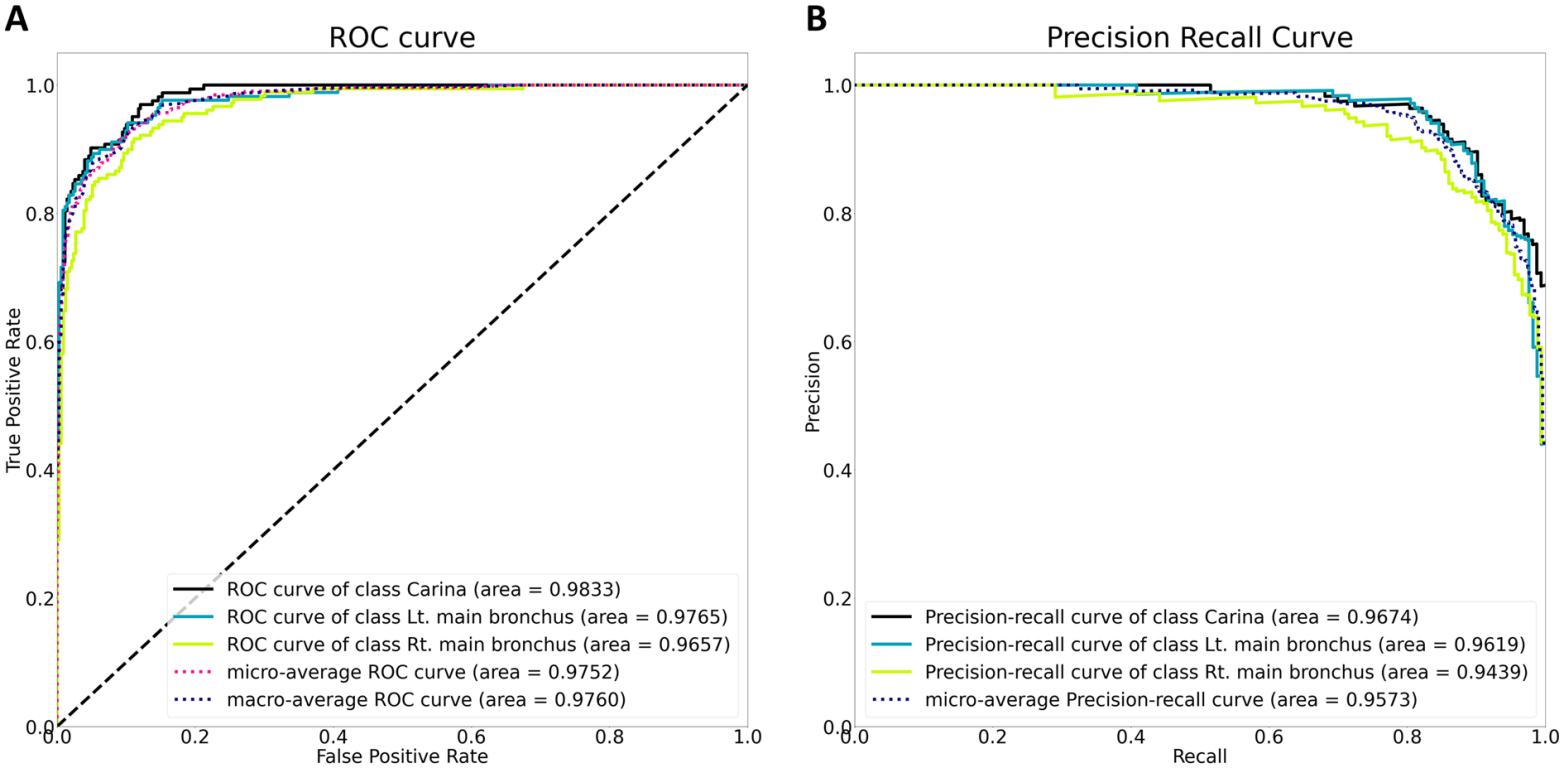
The area under the receiver operating characteristic curve and precision-recall curve of the artificial intelligence model for distinguishing anatomical locations.

With the artificial intelligence model, the AUCs for predicting the carina, left main bronchus, and right main bronchus were 0.9833, 0.9765, and 0.9657, respectively. The class-average AUC was 0.9752. The area under the precision-recall curve for predicting the carina, left main bronchus, and right main bronchus were 0.9674, 0.9616, and 0.9439, respectively. The class-average area under the precision-recall curve was 0.9673 (Fig. 4).

**Figure 5:**
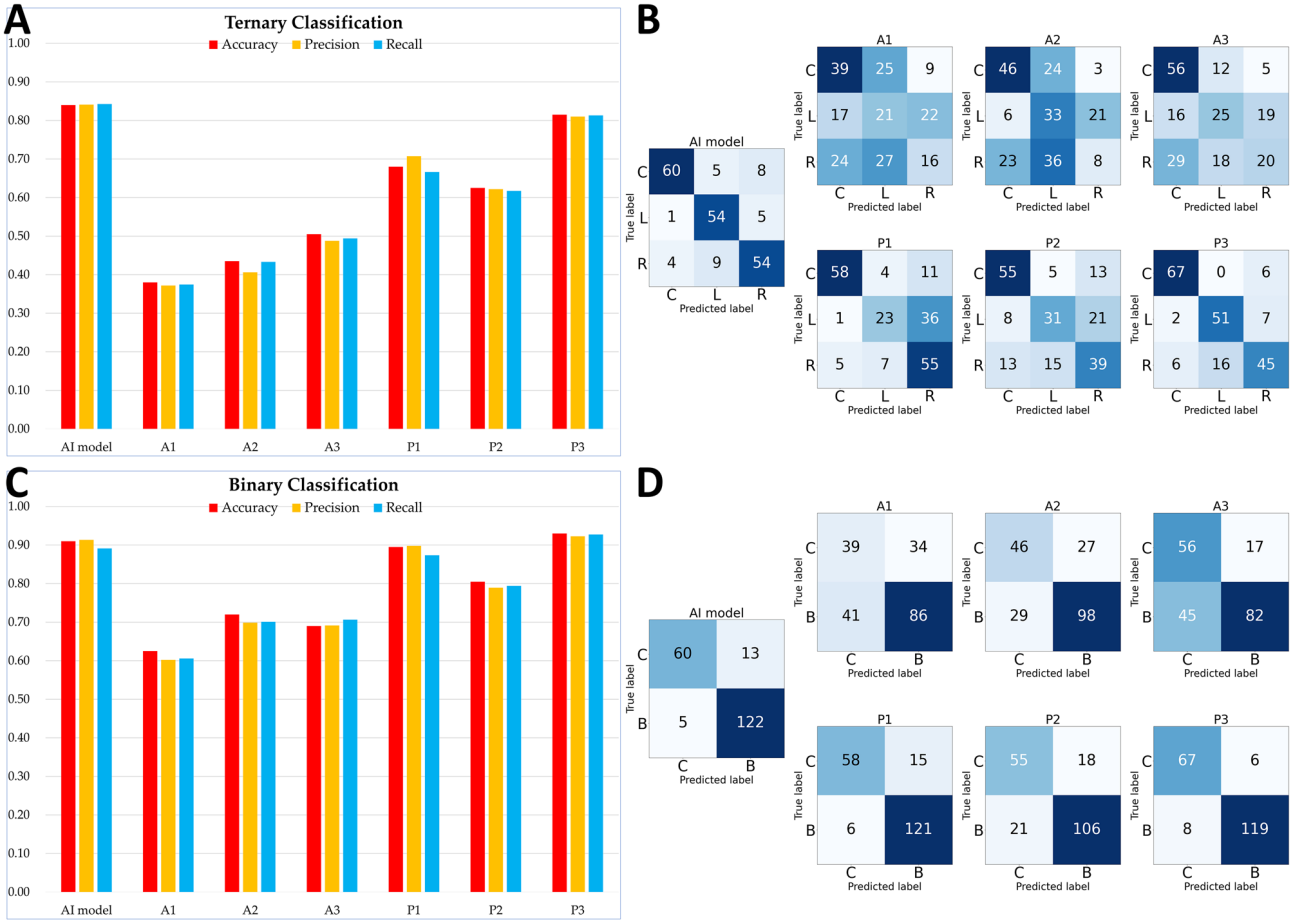
Performance metrics of the artificial intelligence model and human experts. (**A**) Classification metrics for ternary (carina/left main bronchus/right main bronchus); (**B**) confusion matrix for ternary classification; (**C**) classification metrics for binary (carina/both main bronchi); (**D**) confusion matrix for binary classification. A1, A2, and A3 are anesthesiologists with 1, 15, and 24 years of specialist experience and P1, P2, and P3 are pulmonologists with 12, 14, and 20 years, respectively, of specialist experience working in a referral university hospital. Letters C, L, R, and B in the confusion matrix indicate the carina, left main bronchus, right main bronchus, and both main bronchi, respectively.

The performance of the human experts for the evaluation dataset varied. In the ternary classification task, A1 (1 year of anesthesiology specialist experience) showed the lowest accuracy (0.3800) among the human experts, whereas P3 (20 years of pulmonology specialist experience) showed the highest accuracy (0.8150). The accuracy was higher for the artificial intelligence model (0.8400) than for any of the human experts. Except for P3, the performance of the artificial intelligence model was significantly superior. Although the accuracy was slightly lower for P3 than for the artificial intelligence model, the difference was not significant (p = 0.5601). In the binary classification task, the overall results were similar except that P3 outperformed the artificial intelligence model (accuracy 0.9300 vs. 0.9100), although the difference was not significant (p = 0.5572). These results are summarized in Fig. 5. The agreement rate for 20 duplicated but differently rotated and cropped images were 95% (19/20) for the artificial intelligence model and 45%, 65%, 45%, 65%, 70%, and 80% for A1, A2, A3, P1, P2, and P3, respectively.

**Figure 6:**
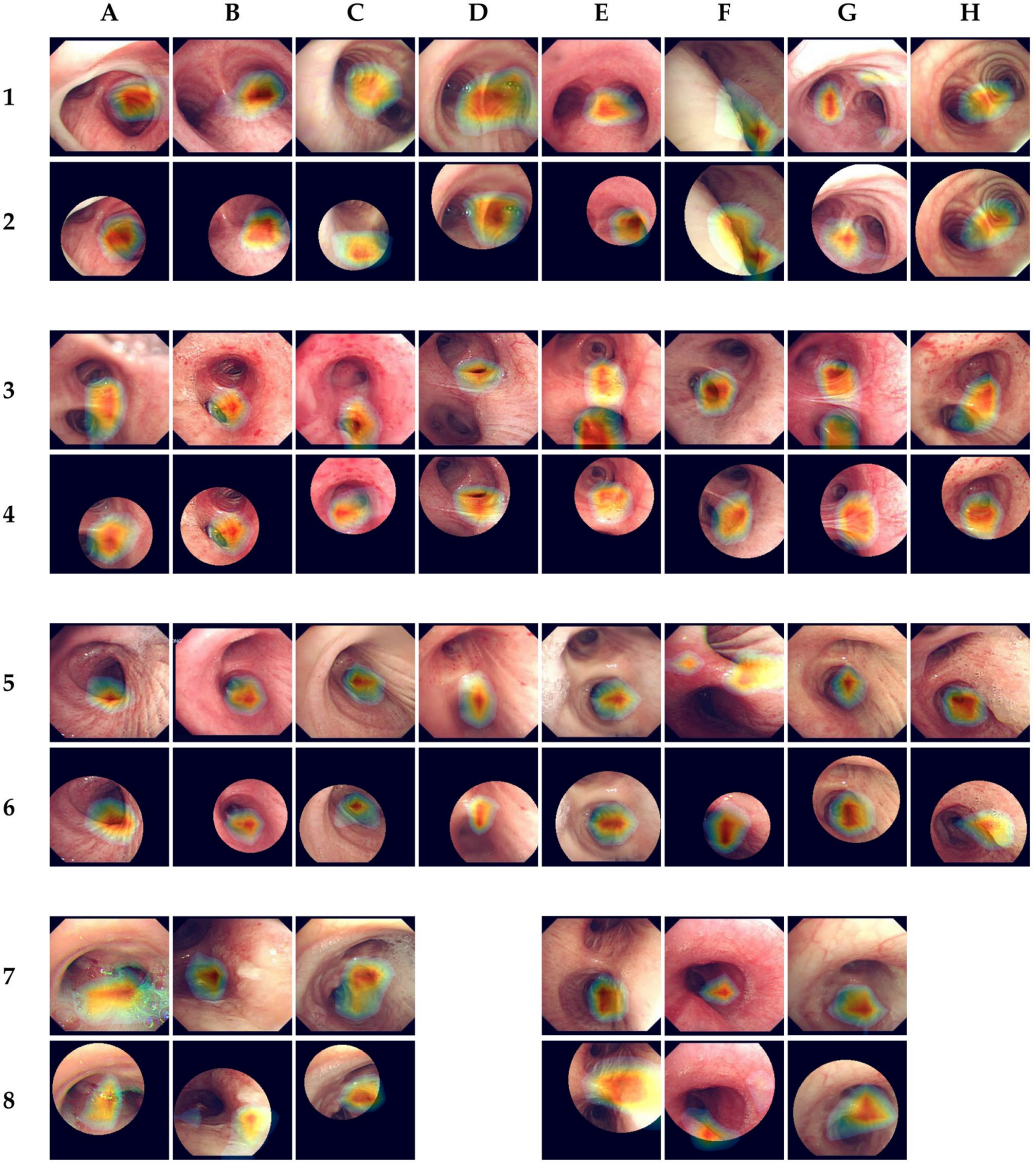
Saliency map generated by gradient-weighted class activation mapping. Odd lines and even lines represent matched original images and circular cropped images, respectively. Images at lines 1 and 2 are correctly predicted cases for the carina and those at lines 3 and 4 are for the left main bronchus. Lines 5 and 6 represent the correctly predicted images of the right main bronchus. Images at lines 7 and 8 indicate the cases in which inference has changed depending on whether the image is cropped. A7, true carina; A8, carina predicted as the right main bronchus; B7, true left main bronchus; B8, left main bronchus predicted as the carina; C7, true right main bronchus; C8, right main bronchus predicted as the left main bronchus; E7, carina predicted as the right main bronchus; E8, true carina; F7, left main bronchus predicted as the right main bronchus; F8, true left main bronchus; G7, right main bronchus predicted as the left main bronchus; G8, true right main bronchus.

Gradient-weighted class activation mapping analysis provided a graphic view of the ability of the artificial intelligence model to predict anatomical locations from video bronchoscopy images (Fig. 6). When the artificial intelligence model predicted the carina, it was mostly focused on the sharp edge of the carina with adjacent bronchial cartilages and posterior muscle stripes. On the other hand, the prediction of the bronchi seemed to be influenced by the features of deeper structures, such as the junction between the secondary and tertiary bronchi. Although almost all circular cropped images showed similar heatmaps for the original images, large differences were noted in some cases with excessive cropping.

## Discussion

In this investigation, our goal was to develop an artificial intelligence model that could anatomically interpret video bronchoscopy images of the carina and main bronchi regardless of rotation or covering. We demonstrated that the classification performance of the artificial intelligence model outperformed that of most human experts and was comparable with that of the most-experienced pulmonologist.

Video bronchoscopy has been an important diagnostic or interventional tool for anesthesiology as well as pulmonary and critical care medicine. Although video bronchoscopy is a safe method, accurate navigation through airways requires thorough training^28,29^. The Accreditation Council for Graduate Medical Education, American College of Chest Physicians, American Thoracic Society, and European Respiratory Society require a certain number of procedures for demonstrating competence in interpretation of examination results^30,31^. However, the training environment can be somewhat more disadvantageous for anesthesiologists than for pulmonologists. Unlike pulmonary video bronchoscopy training, in which the anatomical context is well-perceived through exploration of the trachea and bronchial trees, anesthesiologists often introduce the bronchoscope through the endotracheal tube and directly reach the carina. In 2020, the pulmonologists who participated in this study had each performed an average of 250–300 video bronchoscopies, whereas the anesthesiologists had each performed an average of 80–100 video bronchoscopies in a year. Previous reports demonstrated distinct differences in the procedure lengths and complication rates according to training experiences^32^. Furthermore, with the introduction of video laryngoscopes and supraglottic airways, the number of video bronchoscopies by anesthesiologists has been decreasing^33^. Thus, there are relative differences quantitatively and qualitatively. Hence, we believe our developed model can provide advice comparable with the most-experienced human expert in anatomical location discrimination, which not only enables use in clinical settings but also improves the training process.

Apart from anesthesiologic use, interventional video bronchoscopy, including biopsy, anatomical navigation is more important for targeting appropriate tissue location. Thus, several technologies, including augmented reality and 3D printing, have been used to support video bronchoscopy training^10,28,34,35^. However, those studies required prebronchoscopic computed tomography scan to construct 3D segmented volumes and additional display devices. On the other hand, our artificial intelligence model only needs video bronchoscopic images without any additional examinations to be performed. Moreover, the predicted anatomical location can be overlaid in the same screen that examiners view. In short, our artificial intelligence model can assist examiners directly by predicting the anatomical location of what he or she visualizes around the carina and both main bronchi regardless of rotation and covering in real-time. The mean inference time per image was 44.6 ± 3.1 ms (22.4 images per second) for use of a single V100 GPU and 101.0 ± 6.3 ms (9.9 images per second) for use of an eight-threaded i7 CPU. Considering the small size of the model and short inference time, our artificial intelligence model can be embedded in bronchoscopic equipment without network connections or high-performance GPUs.

Throughout our experiments, the EfficientNetB1 model showed the best performance for the test dataset, whereas the NASNetMobile model showed the worst performance. As shown in Fig. 3, models based on the EfficientNet family showed a relatively steady and continuous increase in accuracy and a decline in losses for both training and validation datasets (Fig. 3A–D). Thus, the overall accuracy of the validation dataset outperformed models based on another pretrained network (Fig. 3C,G). Models based on DenseNets, MobileNetV2, NASNetMobile, and ResNet50V2 showed a relatively early overfitting, and the accuracy of the validation dataset was saturated at < 0.85. The model structure proposed by the innovative scaling method of EfficientNet seemed to be more effective in the learning dataset of this study. The proposed EfficientNet model with compound scaling already showed a tendency to focus on more relevant regions with more object details, whereas other models either lack object details or are unable to capture all objects in other transfer learning tasks^24^. On the other hand, although the NASNetMobile-based model had a larger number of parameters than that of the EfficientNetB0, but it seems that the initial loss value altered and converged to local minimal tasks^24^. On the other hand, although the NASNetMobile-based model had a larger number of parameters than the EfficientNetB0 model, it showed an unstable convergence of loss during early epochs, leading to the highest loss value and lowest accuracy (Fig. 3G,H).

There were several limitations in this study. We collected image data retrospectively to secure a sufficiently large number of images for model training. Although a prospective validation study might be needed for general applications in the medical field, our artificial intelligence model showed excellent performance as assessed using sufficient numbers of separate test and evaluation datasets. Another limitation is that this study included only video bronchoscopic images of the carina and both main bronchi with normal anatomy. Thus, anatomical discrimination along the entire airway could not be trained. To demonstrate the ability to function well as a general clinical decision support system as bronchoscopic assistant, training with more images of various regions and pathological conditions would be needed.

In conclusion, we showed that our artificial intelligence model could identify and distinguish anatomical locations using bronchoscopic images of the carina and both main bronchi with performance comparable with that of the most-experienced pulmonologist, which can overcome various patient position and surrounding tubes. Further studies with various conditional datasets are warranted for developing a general clinical decision support system for video bronchoscopy.
